# A New Method of Rice Moisture Content Determination Using Voxel Weighting-Based from Radio Tomography Images

**DOI:** 10.3390/s21113686

**Published:** 2021-05-26

**Authors:** Nurul Amira Mohd Ramli, Mohd Hafiz Fazalul Rahiman, Latifah Munirah Kamarudin, Latifah Mohamed, Ammar Zakaria, Anita Ahmad, Ruzairi Abdul Rahim

**Affiliations:** 1 Faculty of Electrical Engineering Technology, Universiti Malaysia Perlis, Pauh Putra Campus, Perlis 02600, Malaysia; amira90.ramli@gmail.com (N.A.M.R.); latifah@unimap.edu.my (L.M.); ammarzakaria@unimap.edu.my (A.Z.); 2 Centre of Excellence for Advanced Sensor Technology (CEASTech), Universiti Malaysia Perlis, Pauh Putra Campus, Perlis 02600, Malaysia; latifahmunirah@unimap.edu.my; 3 Faculty of Electronic Engineering Technology, Universiti Malaysia Perlis, Pauh Putra Campus, Perlis 02600, Malaysia; 4 School of Electrical Engineering, Faculty of Engineering, Universiti Teknologi Malaysia, Johor 81310, Malaysia; anita@utm.my (A.A.); ruzairi@utm.my (R.A.R.)

**Keywords:** image reconstruction, moisture measurement, radio tomography, rice moisture content, rice silo

## Abstract

This manuscript presents a new method to monitor and localize the moisture distribution in a rice silo based on tomography images. Because the rice grain is naturally hygroscopic, the stored grains’ quality depends on their level of moisture content. Higher moisture content leads to fibre degradation, making the grains too frail and possibly milled. If the moisture is too low, the grains become brittle and are susceptible to higher breakage. At present, the single-point measurement method is unreliable because the moisture build-up inside the silo might be distributed unevenly. In addition, this method mostly applies gravimetric analysis, which is destructive. Thus, we proposed a radio tomographic imaging (RTI) system to address these problems. Four simulated phantom profiles at different percentages of moisture content were reconstructed using Newton’s One-Step Error Reconstruction and Tikhonov Regularization algorithms. This simulation study utilized the relationship between the maximum voxel weighting of the reconstructed RTI image and the percentage of moisture content. The outcomes demonstrated promising results, in which the weighting voxel linearly increased with the percentage of moisture content, with a correlation coefficient higher than 0.95 was obtained. Therefore, the results support the possibility of using the RTI approach for monitoring and localizing the moisture distribution inside the rice silo.

## 1. Introduction

In Malaysia, rice is a symbol of the local culture, and is consumed daily either as cooked rice or indirectly in the form of rice flour. Despite various initiatives that have been implemented to help expand the national rice production [1], a scenario of increasing demand and declining supply has been forecasted for the next ten years [2,3]. In analysis of the challenges of this agricultural industry, researchers have found that appropriate silo facilities and effective farm management practices should be emphasized to guarantee an adequate supply of domestic production [4,5].

In general, the primary function of a silo is to protect grain crops from any elements, particularly moisture build-up, fungal load, and pest infestation [6,7,8]. However, statistics record that 10–30% of total paddy values (quality and quantity) are degraded due to poor on- and off-farm storage operations [9]. Because the paddy and rice grains are naturally hygroscopic, their value post-harvesting primarily depends on their level of moisture content. The literature has also acknowledged that moisture content is the prominent factor that determines both the proper time for harvesting and the requirement for safe storage conditions [10,11,12].

Following the mechanization of agricultural production, heated forced air dryers were developed to maintain the moisture level inside the silo as a means to preserve the quality and longevity of the grains. Nonetheless, in Malaysia, the storage of rice grains is challenging due to the hot, humid climate, which has a relative humidity of approximately 80%. According to [9,13,14], to preserve the rice quality and allow long-term storage, grains must be dried to 11–14% moisture content. Grains with higher moisture content tend to be frail and may be pulverized, and encourage the development of fungal invasion. In contrast, if the moisture content is too low, the grains become brittle and are exposed to a higher rate of breakage [15,16].

At present, moisture content is usually measured using a straightforward, fast, and convenient moisture meter [17,18]. The sensing application depends on the relationship of the material’s dielectric properties and the frequency of interest [19]. Although this type of instrument provides rapid measurement, it is not practical for moisture sensing of bulk grain in a silo. This failure is due to the single-point measurement, meaning that the measured moisture content does not represent the location and distribution of moisture at the bulk grain or silo scale [20]. Moreover, this method usually applies gravimetric analysis. This destructive process eventually reduces the grains’ quantity throughout the storage period.

Therefore, a comprehensive sensing method is essential to improve the efficiency of moisture distribution assessment in rice silos. Our systematic study presents a simulation of an RTI technique, which can help to visualize the location and percentage of the moisture content. The proposed method focuses on image analysis by employing simulation procedures for data collection, a tomographic technique based on algorithms, and linear regression to evaluate the effectiveness of the proposed method. Consequently, the findings allowed us to explore the feasibility of a tested RTI system as a tool for sensing changes of moisture content, with the aim of establishing a new approach for online, off-site, reliable, and non-invasive sensing methods.

## 2. Methodology

Tomography is a unique approach that is used to reconstruct a cross-sectional image of an object’s internal structure through sensory data obtained by the system [21]. The tomography system does not require invasion of the object of interest (OI), and was designed to analyze the monitored area’s internal composition using penetrating waves to calculate virtual cross-sections. Traditionally, the system’s structure can be visualized as three main components, which are the sensory system, data acquisition, and image reconstruction and display system [22]. The deployed sensor structure acts as the core input to the system, which is built based on the suitability of surroundings, nature of the OI, and the resolution quality of the information required by the user [23,24].

Various sensory systems in tomography applications have been used to localize moisture distribution, for example, electrical capacitance tomography (ECT), electrical resistance tomography (ERT), and microwave tomography (MWT). ECT and ERT are known as electrical tomography, which was developed to visualize the permittivity and conductivity distribution inside the OI by measuring a set of capacitance and resistance between inter-electrodes mounted around the periphery of the OI [25]. In contrast, the MWT technique reconstructs the tomogram based on the measurement of the scattered electromagnetic field from the arrayed sensor nodes [26]. Despite having different sensing elements, researchers have pursued the optimal tomography method to measure the moisture distribution in numerous frameworks.

In the construction industry, previous works as in [27,28] provided a non-destructive tool for approximately imaging the flow, shape, and position of the moisture distribution inside a cement-based material using ERT and ECT techniques. Refrence [29] implemented the ERT system in their geophysics study, based on the detection of moisture availability in the Mediterranean area. In addition, study by [30] found that the ECT system is feasible for imaging the moisture content information in wood. Overall, it can be concluded that, although electrical tomography is laborious, it can successfully deliver the required information about the OI.

However, in the agricultural sector, and particularly for sensing of grain moisture, the MWT system is usually preferred. The MWT system is based on the relationship between electromagnetic behaviour and the material’s relative permittivity [26]. Regardless of the successful application of the MWT technique [31], it is well known to be complicated and costly. This is because the system must be calibrated for dissimilar materials or if more than one variable is encountered in the framework [26].

### Radio Tomographic Imaging (RTI) Approach

**Figure 1:**
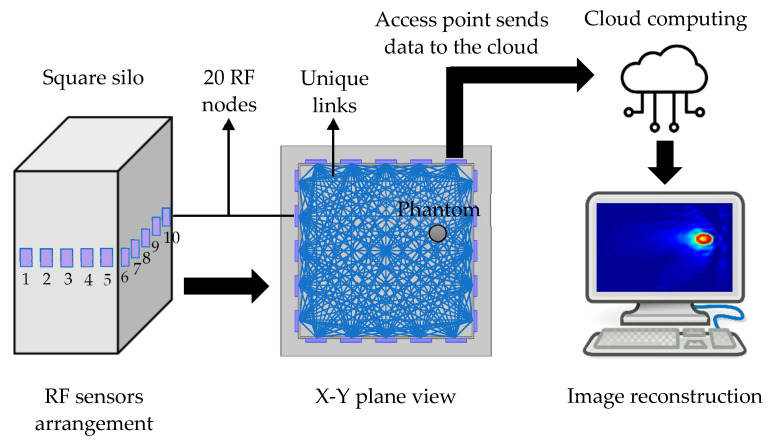
The conceptual view of the RTI system consisting an arrangement of 20 RF nodes, data acquisition process, and image reconstruction system.

As a promising device-free localization technology, the concept of the RTI technique for monitoring and locating targets was initially proposed by [32]. This technology enables the location of targeted objects using image reconstruction based on changes in received signal strength (RSS) of the radio frequency (RF) signals between each stationary sensor node link in a wireless network area [33,34]. It is understood that when an object obstructs the transmission links, the RSS quality of the associated links experiences significant loss, whereas unblocked links are unaffected. Thus, within a monitored RF sensor network, the RTI system determines the targeted object’s location by reconstructing the RSS attenuation map across the sensor network. Figure 1 below illustrates the conceptual view of the RTI system.

In this work, the RTI system was studied in a finite element modelling (FEM) simulation platform. The structure of the geometrical dimension, number of RF nodes, signal frequency, and material dielectric properties were considered thoroughly. The sensor network consists of 20 RF nodes deployed on a conventional 2D silo to test the capability of the RTI technique for moisture distribution sensing in a rice silo. The moisture profiles were reconstructed using appropriate image reconstruction algorithms applied to the entire attenuation maps. The mapping process was relatively simple because it depends on the linearization and weighting model for RTI. In solving the forward problem, the measuring strategy was based on the linearization of its normalized weighted back-projected (Jacobian) matrix from each measurement [25,35].

## 3. Finite Element Modelling of RTI System

**Figure 2:**
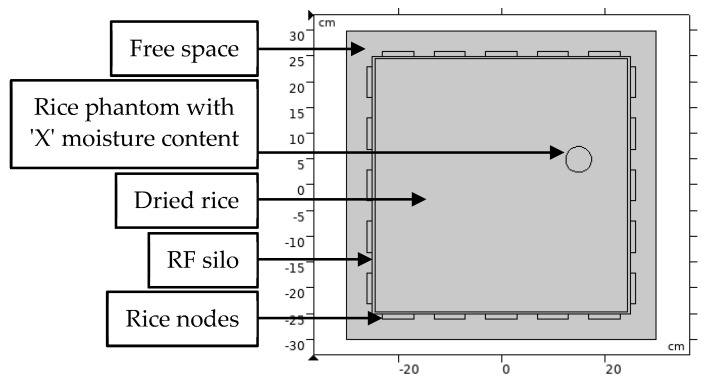
Configuration of the RTI system.

It is essential to study the behaviour of RF energy in a rice medium to control for the confounding variables and ensure the repeatability of obtaining RF attenuation for each scenario in the study. Figure 2 demonstrates the configuration setup of the FEM simulation. The RF sensor network comprising 20 nodes was configured on a 2D square silo. Each node is equally arrayed 40 mm apart, along the perimeter of a 0.5 m × 0.5 m modelled silo with an operating frequency of 2.4 GHz. The 2.4 GHz frequency was chosen to utilize the low-cost and standard ISM band of the wireless sensor network (WSN). The nodes were arranged in such a way to receive the scattered electric fields in multiple directions. The number of pixels that reflect the image’s resolution was set to 300 × 300 pixels. The following subsections comprehensively explain the design and selection of the simulation parameters.

### 3.1. Modelling Electromagnetic Behaviour

The interaction between an electromagnetic wave and the material’s dielectric properties has led to the development of a wide range of practical solutions in remote sensing. The remarkable features of electromagnetic waves when propagating in a dielectric medium include the carrying by the travelling energy of measurable electric (E) and magnetic (H) fields [36,37]. The description of electromagnetism is expressed by Maxwell’s equation below.

(1) Faraday’s Law: ∇×E(r)=−jωB(r)

(2) Ampere’s Law: ∇×H(r)=jωD(r)+J(r)

(3) Gauss’s Law: ∇·B(r)=0

(4) Gauss’s Law: ∇·D(r)=ρ

where E is the electric field intensity $\left(\frac{V}{m}\right)$, H is the magnetic field intensity $\left(\frac{A}{m}\right)$, B is the magnetic flux density (T), D is the electric flux density $\left(\frac{C}{m^{2}}\right)$, J is the electric current density $\left(\frac{A}{m^{2}}\right)$, ρ is the electric charge density $\left(\frac{C}{m^{3}}\right)$, and r indicates the position vector.

These equations depend indirectly on $e^{jwt}$, where $j=\sqrt{-1}$ denotes the imaginary units. The constitutive relations have been applied in the existence of materials in which electromagnetic phenomena take place.

(5) $$D\left(r\right)=\varepsilon_{o}\varepsilon_{r}E\left(r\right)$$

(6) J(r)=σE(r)

(7) $$B\left(r\right)=\mu_{o}\mu_{r}H\left(r\right)$$

where $\varepsilon_{o}$ is the permittivity of free space $\left(\frac{F}{m}\right)$, $\varepsilon_{r}$ is the material’s relative permittivity, $\mu_{o}$ is the permeability of free space $\left(\frac{H}{m}\right)$, and $\mu_{r}$ is the material’s relative permeability.

Below is the derivation of the equation used in the FEM study. Equation (7) is substituted into Equation (1) and curl on both sides, as shown in Equation (8).

(8) $$\nabla \times \left(\nabla \times E\left(r\right)\right)=-j\omega \mu_{o}\mu_{r}\left(\nabla \times H\left(r\right)\right)$$

Substituting Equations (2), (5), and (6) into Equation (8):

(9) $$\nabla \times \left(\nabla \times E\left(r\right)\right)=-j\omega \mu_{o}\mu_{r}\left(j\omega \varepsilon_{o}\varepsilon_{r}E\left(r\right)+\sigma E\left(r\right)\right)$$

(10) $$\nabla \times \left(\nabla \times E\left(r\right)\right)=\omega^{2}\mu_{o}\mu_{r}\varepsilon_{o}\varepsilon_{r}E\left(r\right)-j\omega \mu_{o}\mu_{r}\sigma E\left(r\right)$$

Equation (10) is simplified into Equations (11) and (12) as below.

(11) $$\nabla \times \left(\nabla \times E\left(r\right)\right)=k_{o}^{2}\mu_{r}\left(\varepsilon_{r}-j\left(\frac{\sigma }{\varepsilon_{o}\omega }\right)\right)$$

(12) $$\nabla \times \mu_{r}^{-1}\left(\nabla \times E\left(r\right)\right)=-k_{o}^{2}\left(\varepsilon_{r}-j\left(\frac{\sigma }{\varepsilon_{o}\omega }\right)\right)$$

where $k_{o}=\omega \sqrt{\varepsilon_{o}\mu_{o}}$ is the wavenumber in free space.

### 3.2. Modelling and Simulation: Rice Grains

As discussed above, the complex dielectric properties of rice grains, $\varepsilon_{r}$, are the parameters that vary significantly with exposed electromagnetic waves. Dielectric properties, or permittivity, are the electrical characteristics of poorly conducting materials. Their dependence on the material’s moisture content (MC) has been of interest due to their practicality for rapid moisture sensing [38]. When interacting with the RF, these properties are studied as the parameters that can be polarized by the electric field. Thus, this RTI system is based on the relationship between electromagnetic energy and the medium’s dielectric properties. The relative complex permittivity of the rice is expressed in Equation (13) [19].

(13) $$\varepsilon_{r}=\varepsilon^{\prime }-j\varepsilon^{\prime \prime }$$

where $\varepsilon^{\prime }$ is the dielectric constant that describes the capability of a material to store energy in the electric field, and $\varepsilon^{\prime \prime }$ is the dielectric loss factor that indicates the capability of a material to dissipate energy from the electric field, which is then converted into heat energy.

Furthermore, the conductivity of the material’s dielectric σ cannot be neglected, and is defined by Equation (14).

(14) $$\sigma =\omega \varepsilon_{o}\varepsilon^{\prime \prime }$$

where ω=2πf is the angular frequency with f in Hz, and $\varepsilon_{o}$ is the permittivity of free space, which is equivalent to 8.854 $\times 10^{-12}$ F/m.

The nature of the correlation between dielectric properties and changes in MC of rice grains is well known [19,39]. Apparently, at any applied frequency, the dielectric constant, $\varepsilon^{\prime }$, is almost linearly correlated with rice grain MC. The loss factor, $\varepsilon^{\prime \prime }$, has been found to be less predictable, and may increase or decrease, depending on the particular range of MC.

The modelling of the rice grains was separated into two parts, which are the dried rice as a reference medium and the phantoms that indicate the rice with ‘X’ MC. The parameters for both conditions, such as dielectric properties, size, and shape, were defined with certanity to understand the ordinary deterministic electromagnetic scattering. Because the nature of moisture distribution is unpredictable [40], this initial study presents a simple geometric shape for the rice phantoms using a built-in primitive object. This commonly used shape can be modified based on the desired dimension and position.

The dried rice medium was assumed to be at 14% MC based on the standard level of MC for rice storage following the drying process. For the rice phantoms, the MC was set incrementally by 2% MC, starting from 16% up to 28%. The upper value of 28% MC was chosen to demonstrate the worst condition for rice grains, because the maximum MC of paddy grains post-harvesting process is about 25% [9]. Equations (15) and (16) below represent the correlation between complex dielectric properties of rice grains, $\varepsilon^{\prime }$ and $\varepsilon^{\prime \prime }$, with MC, based on the regression method modelled by [41,42,43], using the average of ten common types of rice grain in Malaysia’s marketplace.

(15) $$\varepsilon^{\prime }=a_{0}^{\prime }+a_{1}^{\prime }MC+a_{2}^{\prime }MC^{2}$$

(16) $$\varepsilon^{\prime \prime }=a_{0}^{\prime \prime }+a_{1}^{\prime \prime }MC+a_{2}^{\prime \prime }MC^{2}$$

where the value of coefficients $a_{0}^{\prime },a_{1}^{\prime },a_{2}^{\prime }$ and $a_{0}^{\prime \prime },a_{1}^{\prime \prime },a_{2}^{\prime \prime }$ are for the polynomial expression of the dielectric constant, $\varepsilon^{\prime }$, and dielectric loss factor, $\varepsilon^{\prime \prime }$, respectively.

The coefficients in Equations (15) and (16) are expressed as a function of frequency, f, as tabulated in Table 1 below [41,42,43].

The particular electrical conductivity, σ, for the dry medium and rice phantom ‘X’ MC, was calculated using Equation (14). In this simulation study, the temperature was presumed and fixed at 26 °C, because a change in temperature causes the grains’ dielectric properties to vary slightly [44]. In addition, in Malaysia, to preserve the quality of paddy and rice grains, the domestic crops are temporarily stored in warehouses under an ambient temperature range between 26 and 28 °C [3].

### 3.3. Modelling and Simulation: Rice Silo

Based on Figure 2, the rice silo was designed as a square shape made of an acrylic plate with a wall thickness of 10 mm. Because the scattering mechanism may include wave diffraction, the silo size was considered to be sufficiently substantial relative to the wavelength of the propagating frequency to avoid diffraction of the electromagnetic waves between the sensor nodes [45]. However, the attenuation of the electromagnetic waves increases with the increasing size of the medium [46].

As mentioned above, the dimensions of the silo used in this simulation study were 0.5 × 0.5 m. These dimensions were specifically chosen to investigate the capability of the RTI technique to image the moisture distribution inside the silo. The dielectric properties of the silo material were chosen from the predefined FEM input model. Polymethyl methacrylate (PMMA) with relative permittivity and conductivity of 2.5 and 0.0068 S/m, respectively, was selected due to its popularity in various applications [47]. In addition, the same material will be used for our future experimental study.

### 3.4. Modelling and Simulation: RF Nodes

It is requisite to have a mathematical model for the RF sensor nodes so that the RTI system can accommodate simulation of silos of any size with a flexible number of sensor nodes. In this study, we considered a rectangular waveguide design for the RF nodes operating at 2.4 GHz and TE_10_ mode. To apply a scalar model for the RTI problem, it was assumed that under the TE_10_ mode, an incident wave is polarized along the *z*-axis where $E\left(x,y,z\right)=E\left(x,y\right)e^{-ik_{z}z}$, which signifies that $E_{x}$ and $E_{y}$ are equal to zero [48]. The width of the sensor node, w, was modelled and calibrated through Equation (17) [48].

(17) $$f_{c}=\frac{c_{o}}{2w\sqrt{\varepsilon_{r}}}$$

where $f_{c}$ is the cut-off frequency depending on the width of the waveguide, w. $c_{o}$ is the speed of light in a vacuum, which is equivalent to 3 $\times \;10^{8}$ ms^−1^. If the cut-off frequency is lower than the operating frequency, RF signals can propagate in the dried rice medium.

According to [49], in TE_10_ mode, the cut-off frequency must be higher than 1.22 GHz and less than 2.45 GHz. Herein, the width of the sensor node, w, was 60 mm at a cut-off frequency of 1.31 GHz. In addition, the node density also plays a key role in the accuracy of the RTI results. In the same area of geometry, the imaging result is expected to be more accurate where nodes are placed more closely together than in areas where the nodes are spaced at a larger distance [35,50]. Our FEM simulation used 20 RF nodes in the square network within an imaging area of 0.25 m^2^. Although a greater number of nodes could attain better images, we considered the size of the silo.

Furthermore, the transmission power was restricted to 1 mW. When dealing with electromagnetic energy, heating of dielectric materials is subject to the material density and the power source [37]. Therefore, the designated transmission power should be carefully considered, because it may lead to changes in the physicochemical properties of the rice in practice [51].

## 4. Solving Forward Problem

### 4.1. Measuring Strategy

The basic linear formulation model was applied to determine the scattered electric field measurement values for known attenuation coefficients on each discretized pixel weight. The discretized electric field was solved based on the distribution of dielectric properties of the dried rice, rice phantoms, and rice silo. This technique is accessible because the dielectric properties are capable of manipulating the behaviour of the electric field.

In the RTI system, the electric field was excited within the network region through the rice phantom with ‘X’ MC. An array of 20 RF nodes were utilized to determine the signal strength from each transmitting and receiving node. The transmission links between node 1 and 2, and between node 2 and 1, in the network area are the same because any pair of nodes is regarded as a link, regardless of whether communication exists between them. Therefore, if K is the number of sensor nodes deployed in the RTI system, then the total number of unique two-ways links, M, is expressed as Equation (18) [32].

(18) $$M=\frac{K^{2}+K}{2}$$

If node 1 is assigned to function as transmitter, then the remaining nodes, 2, 3, 4, …, K=20, are allocated to serve as receivers. The procedure was sequentially repeated until node K=20 was transmitting, and nodes 1 to 19 were receiving. Hence, with a K number of 20 nodes, the maximum number of measured radio links, M, would be M=190.

### 4.2. Weighting Model

From the local linearization, 190 sensitivity maps, also known as the Jacobian matrix, J, were computed from the electric field strength distribution, E(x,y). One element, $J_{ji}$, of this matrix defines the electric field, E(x,y), and changes at a measurement area due to a slight perturbation in the dielectric properties of one element, $\varepsilon_{r}\left(x,y\right)$, of the model [48]. This is stated as in Equation (19).

(19) $$J_{ji}=\frac{\partial E\left(x,y\right)}{\partial \varepsilon_{r}\left(x,y\right)}$$

**Figure 3:**
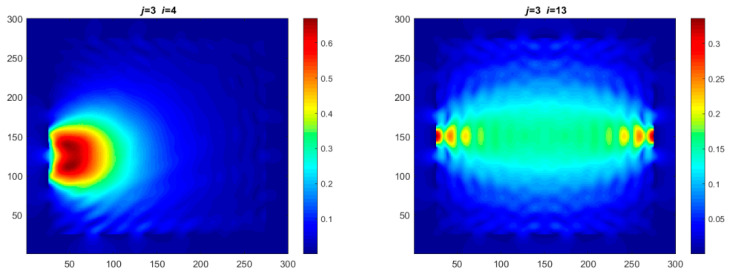
Normalized sensitivity maps of excitation at node, *j* = 3, with corresponding measurement at node *i* = 4 and *i* = 13.

Each Jacobian matrix was derived from the FEM mesh of the forward model without the elements established in the dried rice (reference medium) and RF nodes. In this study, the electric field distribution was computed on the 11,000 finer tetrahedral mesh. The grid size for each Jacobian matrix was 0.002 m, and the number of grid pixels in the computational domain was 300 × 300. Figure 3 shows the normalized sensitivity maps of excitation at RF nodes, j=3, with the corresponding adjacent and opposite measurement at RF node i=4 and i=13, respectively. It can be seen that, as the distance between two nodes increases, the sensitivity decreases.

In the RTI technique, the signals’ attenuation due to the phantom’s presence can be integrated by uniformly divided the imaging area into many small square pixels, N [52]. Because the attenuation of a link contributes differently to each pixel value, a weighting model was applied. Therefore, in this simulation study, the weight balanced map could be approximated as the sum of multiplication of the Jacobian matrix, $J_{ji}$, by its corresponding signal loss, $L_{ji}$, as shown in Equation (20).

(20) $$W_{N}=\overset{T}{\underset{j=1}{\sum }}\overset{R}{\underset{i=1}{\sum }}L_{ji}\times J_{ji}$$

where $L_{ji}=S_{ij}^{tot}-S_{ij}^{inc}$, T denotes the number of excitation nodes, R denotes the number of measuring nodes, $S_{ij}^{tot}$ is the electric field measured when there is a rice phantom with ‘X’ MC, and $S_{ij}^{inc}$ is the electric field measured before the presence of the rice phantom.

## 5. Solving Inverse Problem

Image reconstruction aims to solve the inverse problem by generating the real image, which determines the unknown electric field distribution vector in the FEM domain. Although this technique does not provide precise information about the phantom’s location (moisture distribution), the electric field does represent some changes in the distribution of the different dielectric properties, which later enable reconstruction of rice phantoms [22]. This study applied two qualitative imaging algorithms, namely Newton’s One-Step Error Reconstruction (NOSER) and Tikhonov Regularization.

### 5.1. NOSER Algorithm

NOSER is a fast and practical image reconstruction algorithm due to its stability and direct linear method [53]. The formulation of NOSER in this RTI system is as shown in Equation (21).

(21) $$G_{NOSER}\left(x,y\right)={\left[H\right]}^{T}\times \overset{T}{\underset{j=1}{\sum }}\overset{R}{\underset{i=1}{\sum }}LS_{ji}\times J_{ji}\left(x,y\right)$$

(22) $$\mathrm{where}\;LS_{ji}={\left[S_{ij}^{tot}-S_{ij}^{inc}\right]}^{2}$$

(23) $$\mathrm{and}\;H=J_{ji}\left(x,y\right)*{\left[J_{ji}\left(x,y\right)\right]}^{T}$$

where H is the Hessian matrix and $J_{ji}\left(x,y\right)$ is the computed Jacobian matrix corresponding to its signal loss, $L_{ji}$ as described above.

$L_{ji}$ is calculated using the least square method. The least square method aims to minimize the variation between the $S_{ij}^{inc}$ and $S_{ij}^{tot}$ for a given electric field distribution in the computational domain. In addition, the transposed Hessian matrix is utilized as a rough approximation instead of using inverse because it is impossible to compute the direct inverse [54].

### 5.2. Tikhonov Regularization Algorithm

Tikhonov Regularization is a commonly used method that provides a simple framework for integrating desired characteristics into the RTI reconstruction. In this study, the summation of the Hessian matrix was decomposed into three constituent matrices, which were U, V, and Σ. U and V are the unitary matrices, and Σ is a diagonal matrix. The diagonal elements of Σ are singular values, ρ, as expressed in Equation (24).

(24) ρ=diag(Σ)

Using Equation (23), regularization involves introducing additional information into the mathematical model to handle these small singular values and stabilize the inverse problem [55].

(25) $$T_{tikh,\kappa }=\frac{\rho }{\rho^{2}+\kappa }$$

where κ is the regularization parameter and κ>0.

The formula to reconstruct the image using the Tikhonov Regularization algorithm is shown in Equation (26) for this RTI system.

(26) $$G_{tikh}\left(x,y\right)=U\times V\times diag\left(T_{tikh,\kappa }\right)\times \overset{T}{\underset{j=1}{\sum }}\overset{R}{\underset{i=1}{\sum }}LS_{ji}\times J_{ji}\left(x,y\right)$$

## 6. Results and Discussion

The image reconstruction and analysis were based on the pixel values. These pixel values represent the density related to the scattered electric field distribution inside the imaging area. In this section, we discuss the imaging results of the static moisture phantoms, which involved different MC percentages, investigate the approximation of MC percentage based on the regression plot of maximum pixel values, and examine the feasibility of the developed RTI system for rice MC imaging. We include an assessment of image quality based on different image reconstruction algorithms, i.e., NOSER and Tikhonov Regularization.

The results of all reconstructed images were quantified using a Mean Structural Similarity Index (MSSIM) image quality assessment by comparing the reconstructed image with the reference image. The evaluation aimed to analyze the similarity between the two images in terms of structure, luminance, and contrast, which gives an output index in the range of 0 to 1 [56]. An output index approaching one indicates the reconstructed image is close to the reference image.

**Figure 4:**
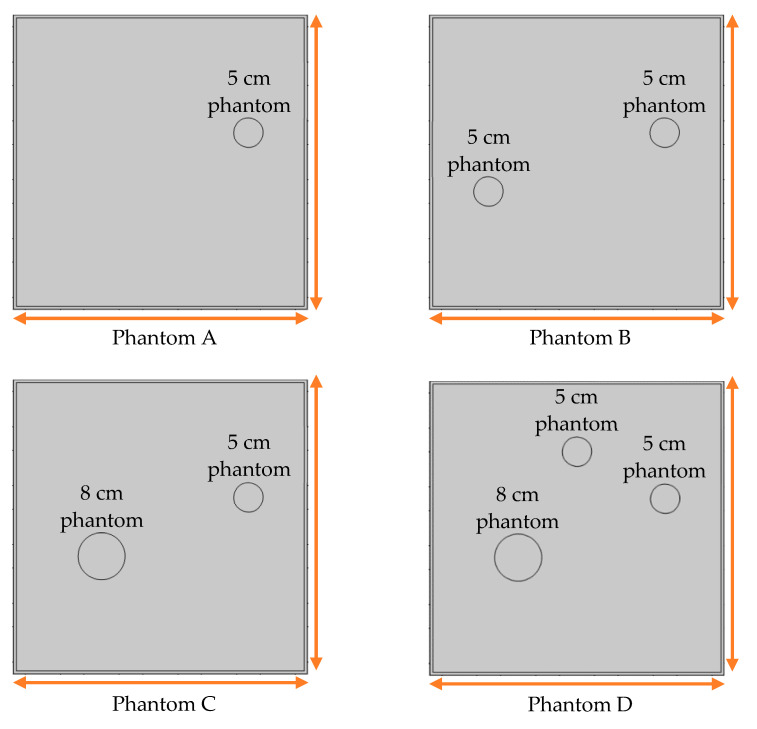
Simulation of rice phantom (moisture distribution).

The FEM simulation studies were conducted using the selected frequency, phantom properties, and background values. Four different scenarios of moisture distribution with seven different sets of MC percentage each were considered to evaluate the performance of the RTI concept, as shown in Figure 4. The reconstructed images using NOSER and Tikhonov Regularization algorithms for Phantoms A, B, C, and D are presented in Table 2 and Table 3, where the highest and lowest constrasts are indicated in red and blue, respectively.

Using the Tikhonov Regularization algorithm, different regularization parameters yield different tomogram results. Therefore, the chosen parameter should provide good results based on the ability to distinguish the changes in electric field attenuation and the limitation of the iteration number. In this study, the regularization parameter, κ of $\kappa =10^{-16}$, was selected as the optimum parameter to reconstruct the Phantoms A, B, C, and D at MC percentages of 16%, 18%, 20%, 22%, 24%, 26%, and 28%.

Table 2 and Table 3 illustrate that the simulation results exhibit successful image reconstruction, which supports the possibility of using RTI to monitor and localize the moisture distribution in a rice silo. The rice phantoms reconstructed using both NOSER and Tikhonov Regularization algorithms possessed a position corresponding to the original simulated phantoms’ profiles for seven different MC percentages. Despite the presence of smearing-effect artefacts, the phantom shape and position can be rocognized and distinguished from the dried rice context.

Although both NOSER and Tikhonov Regularization approaches applied the least square method, NOSER does not perform an iterative procedure, which leads to shorter reconstruction time. Tikhonov Regularization iteratively involves additional regularization parameters into the mathematical model, thus required extra calculation time. In general, we noticed that the image reconstruction results using Tikhonov Regularization were preferable to those of NOSER, for which the regularization leads to smoothing of the reconstructed profiles. Hence, the selection of the algorithm should be task-specific.

### 6.1. Analysis of Maximum Pixel Value

Before the measurement and image reconstruction procedures, the reference medium was represented by dried rice at its respective dielectric properties, and the reference measurement was conducted. Then, a different permittivity of rice phantom at ‘X’ MC was introduced into the reference medium. The reconstruction algorithm was used to analyze the difference between the pixels’ density data for the scenario with and without phantoms, and to calculate a qualitative approximation of the permittivity distribution. Therefore, the maximum (max) weighting pixel of the reconstructed images should indicate its corresponding ‘X’ MC.

Based on this understanding, we utilized the max pixel value from the reconstructed image’s grid to form a linear regression plot. Because NOSER and Tikhonov Regularization algorithms offered different baselines of the max pixel value, the data was quantified using the percentage of the max pixel value from the tomogram of the reference medium in which the rice phantom was absent. As in the dried rice medium, the max pixel value was equivalent to 16.89. After the calculation, the max pixel value in terms of percentage for both algorithms can be appropriately compared.

**Figure 5:**
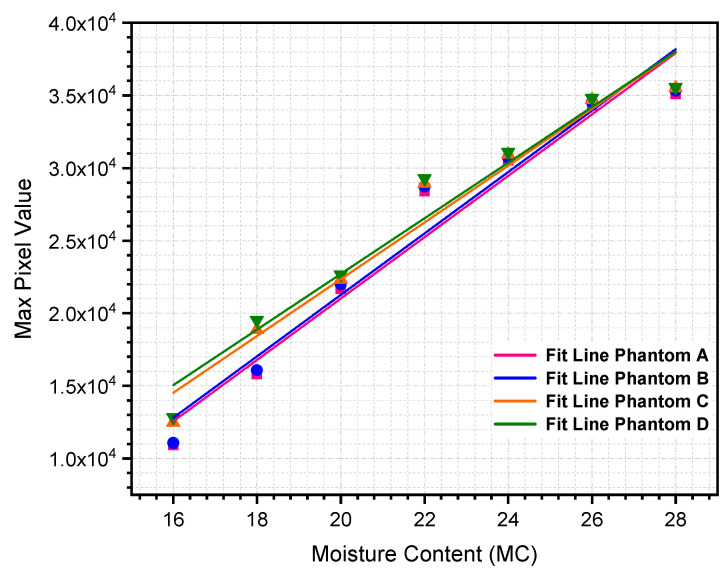
Percentage of maximum pixel value using NOSER algorithm.

**Figure 6:**
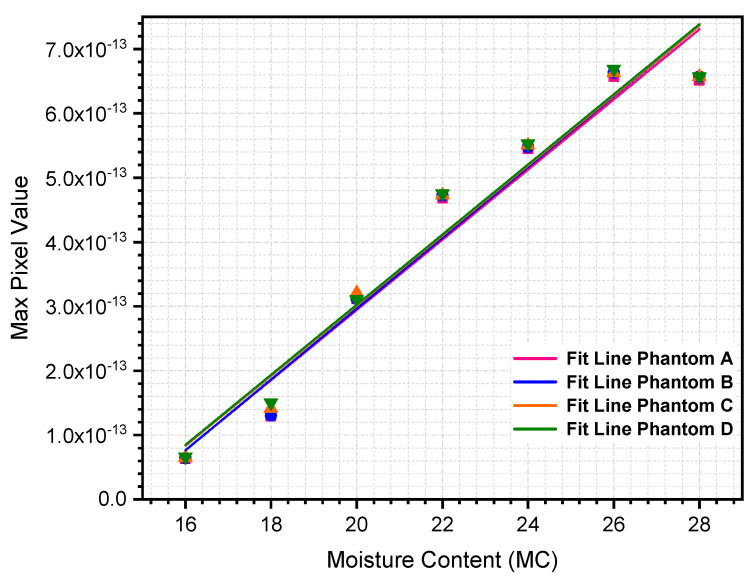
Percentage of maximum pixel value using Tikhonov Regularization algorithm.

Table 4 and Table 5 below show the percentage of max pixel value as a function of MC using NOSER and Tikhonov Regularization for MC at 16% to 28%, respectively. The tabulated data are plotted, as depicted in Figure 5 and Figure 6.

All of the reconstructed images in Table 2 and Table 3 show the existence of rice phantoms with ‘X’ MC by indicating changes in the color scale. However, these tomograms only exhibit positive results on the location and size of the moisture distribution. In addition, due to the smearing effects, it was difficult to determine the changes in terms of MC intensity. Therefore, the analyses, as illustrated in Figure 5 and Figure 6 are at least measurable.

The relationship between max pixel value and rice MC percentage can be quantitatively reconstructed using a linear regression approach. Both reconstruction algorithms, NOSER and Tikhonov Regularization, indicated that the max pixel value increases steadily as the rice MC increases. Because the max pixel value increases linearly with the percentage of MC, a first-order polynomial regression model was preliminary predicted, as shown in Equation (27).

(27) $$PV_{max}=aMC-b$$

where a and b are the coefficients specific to each regression.

The statistical regression analyses are summarized in Table 6 and Table 7 for NOSER and Tikhonov Regularization algorithms, respectively. Based on the corresponding tables, the results indicate that the percentage of rice MC is the most significant parameter affecting the max pixel value, with a correlation coefficient, $R^{2}$, higher than 0.95 regardless of the location and size of the rice phantoms used in this study.

Although the correlation coefficient, $R^{2}$, results using the NOSER algorithm were slightly higher than those of the Tikhonov Regularization algorithm, we concluded that the Tikhonov Regularization is the preferred approach because it yielded the most stable linear regression model, as seen in Figure 5 and Figure 6. It is clear that the ill-posed inverse problem of RTI is stabilized by introducing a regularization parameter into the mathematical framework.

### 6.2. MSSIM Analysis

**Figure 7:**
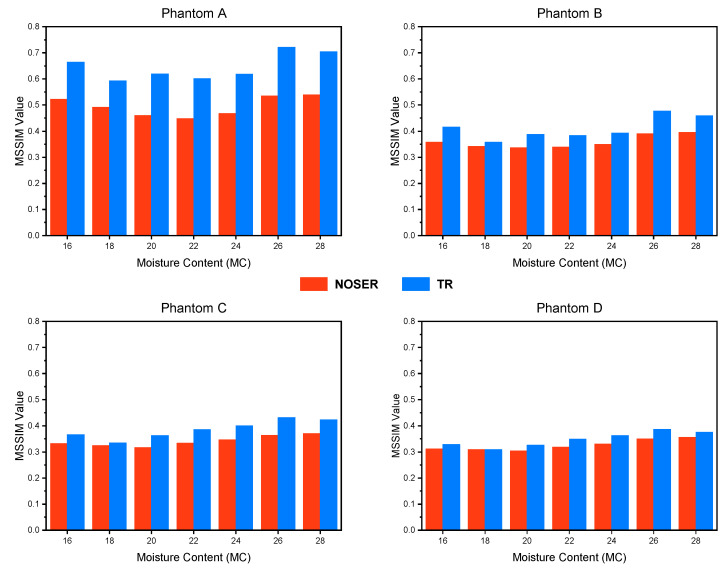
MSSIM Index graphs computed on the reconstructed images of Phantom A, B, C, and D.

To quantitatively compare different algorithms, the MSSIM Index was calculated for the reconstructed images concerning the real distribution. The data is tabulated in Table 8 and presented in Figure 7.

As presented in Figure 7, we can observe that the Tikhonov Regularization algorithm recorded the highest MSSIM Index for all four moisture distribution profiles. The highest and lowest MSSIM Indexes scored by the Tikhonov Regularization algorithm were 0.7211 for Phantom A at MC of 26%, and 0.3092 for Phantom D at MC of 18%, respectively. For the MSSIM Index scored by the NOSER algorithm, the highest and lowest indexes were 0.5389 for Phantom A at MC of 28%, and 0.3040 for Phantom D at MC of 20%, respectively. Therefore, it can be deduced that the Tikhonov Regularization technique is better in reconstructing the moisture distribution in the rice silo.

Overall, moderate MSSIM indexes were obtained by the RTI system. This might be attributed to the difference in dielectric properties of dried rice and rice with ‘X’ MC, which causes a relatively small re-polarization (to the initial field) of an incident field, $E^{inc}$. However, the results demonstrate the possibility of implementing the RTI system for rice moisture monitoring and localization.

## 7. Conclusions

In conclusion, monitoring and localizing the moisture distribution in a rice silo is essential in the agricultural industry to ensure the sustainability of domestic production. In this preliminary study, the feasibility of an RTI system for rice moisture detection was successfully simulated, and the analysis of its performance was appropriately carried out. The comprehensive studies on the proposed FEM simulation data confirmed the findings and ensure that the results were not artefacts due to simulation errors.

The results indicate that the percentage of rice MC is a parameter that has a significant effect on the max pixel value, with a correlation coefficient, $R^{2}$, higher than 0.95. Therefore, this study provides evidence to support the possibility of using the RTI technique for monitoring the rice moisture distribution, based on the study of maximum pixel values from the reconstructed tomogram. Furthermore, we also evaluated MSSIM Indexes to quantitatively compare the two algorithms. The findings showed that both NOSER and Tikhonov Regularization algorithms performed good image reconstruction with moderate MSSIM Indexes.

Our assessments presented in this paper demonstrate that the Tikhonov Regularization algorithm possessed a more stable technique in monitoring and localizing the rice moisture distribution in the RTI system. This stability is due to the incorporation of the regularization parameter into the mathematical model. Nevertheless, the results of this initial study could perhaps serve as a non-destructive tool to monitor and localize the moisture distribution in rice silos.

Finally, regarding an actual case study, further research work should be conducted to explore the reliability, sensitivity, and effectiveness of the proposed image reconstruction technique. It is recommended that future studies focus on the development of pre-processing of the sensory data [57,58] because the reconstructed images may be affected by the intrinsic behavior of the images due to factors related to noise and the environment.

## Figures and Tables

**Table 1:** Quadratic coefficients of Equations (15) and (16).

Coefficient Functions	
Dielectric constant, $\varepsilon^{\prime }$	$a_{0}^{\prime }=-0.4605f^{2}+8.5289f-5.1708$
	$a_{1}^{\prime }=0.0544f^{2}-0.9928f-0.2982$
	$a_{2}^{\prime }=-0.0016f^{2}+0.0281f+0.0224$
Dielectric loss factor, $\varepsilon^{\prime \prime }$	$a_{0}^{\prime \prime }=0.1347f^{2}+0.6010-8.4663$
	$a_{1}^{\prime \prime }=-0.0166-0.0693f+0.8215$
	$a_{2}^{\prime \prime }=0.0005f^{2}+0.0023f-0.0149$

**Table 2:** Reconstructed images for Phantoms A and B at different percentages of MC.

MC (%)	Phantom A		Phantom B		
	NOSER	Tikhonov Regularization	NOSER	Tikhonov Regularization	
16	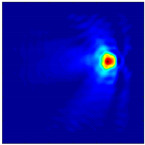	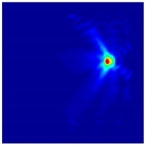	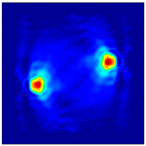	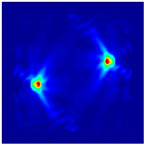	High
18	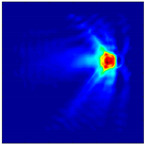	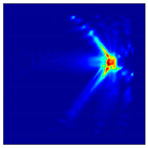	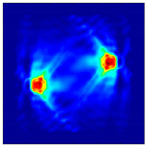	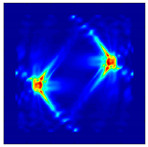	** 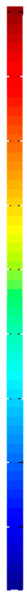 **
20	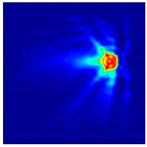	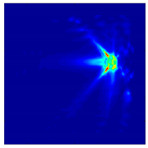	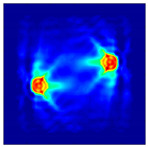	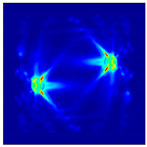	
22	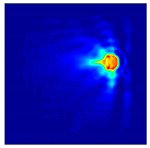	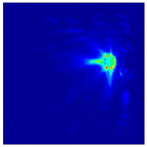	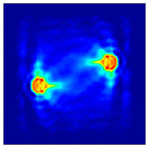	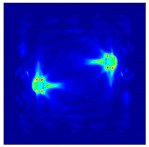	
24	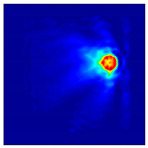	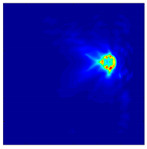	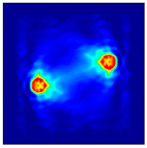	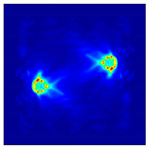	
26	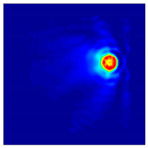	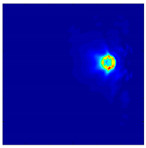	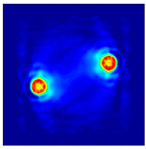	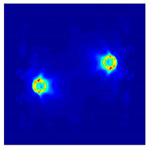	
28	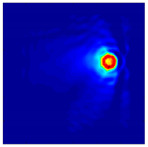	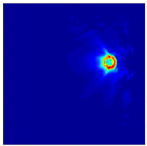	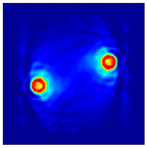	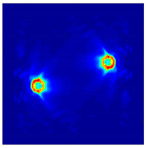	Low

**Table 3:** Reconstructed images for Phantoms C and D at different percentages of MC.

MC (%)	Phantom C		Phantom D		
	NOSER	Tikhonov Regularization	NOSER	Tikhonov Regularization	
16	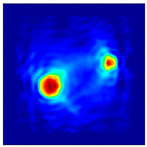	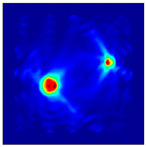	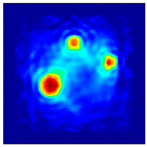	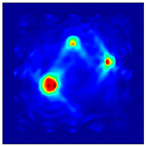	High
18	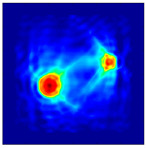	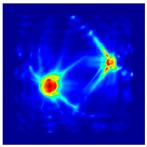	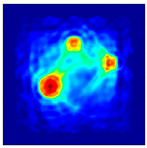	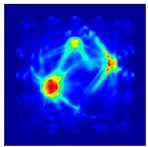	** 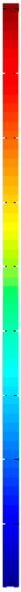 **
20	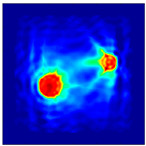	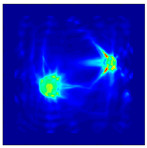	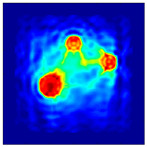	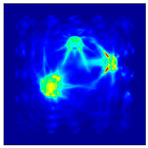	
22	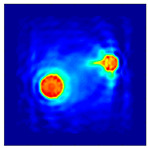	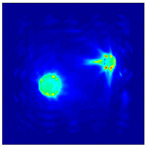	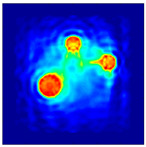	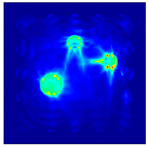	
24	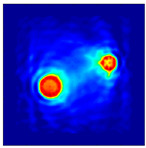	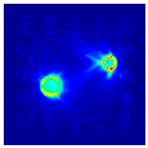	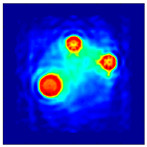	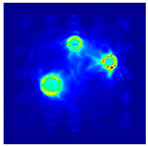	
26	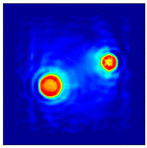	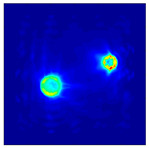	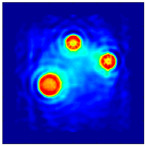	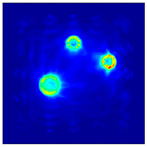	
28	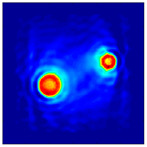	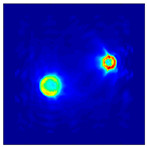	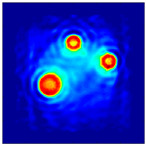	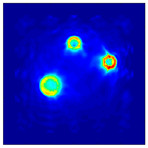	Low

**Table 4:** Percentage of maximum pixel value using NOSER algorithm.

MC (%)	Phantom A	Phantom B	Phantom C	Phantom D
16	$1.092\times 10^{4}$	$1.107\times 10^{4}$	$1.248\times 10^{4}$	$1.283\times 10^{4}$
18	$1.579\times 10^{4}$	$1.607\times 10^{4}$	$1.886\times 10^{4}$	$1.954\times 10^{4}$
20	$2.167\times 10^{4}$	$2.199\times 10^{4}$	$2.233\times 10^{4}$	$2.266\times 10^{4}$
22	$2.840\times 10^{4}$	$2.873\times 10^{4}$	$2.893\times 10^{4}$	$2.927\times 10^{4}$
24	$3.053\times 10^{4}$	$3.074\times 10^{4}$	$3.096\times 10^{4}$	$3.110\times 10^{4}$
26	$3.429\times 10^{4}$	$3.454\times 10^{4}$	$3.471\times 10^{4}$	$3.482\times 10^{4}$
28	$3.509\times 10^{4}$	$3.533\times 10^{4}$	$3.552\times 10^{4}$	$3.558\times 10^{4}$

**Table 5:** Percentage of maximum pixel value using Tikhonov Regularization algorithm.

MC (%)	Phantom A	Phantom B	Phantom C	Phantom D
16	$6.334\times 10^{-14}$	$6.393\times 10^{-14}$	$6.542\times 10^{-14}$	$6.630\times 10^{-14}$
18	$1.290\times 10^{-13}$	$1.302\times 10^{-13}$	$1.415\times 10^{-13}$	$1.540\times 10^{-13}$
20	$3.114\times 10^{-13}$	$3.114\times 10^{-13}$	$3.208\times 10^{-13}$	$3.108\times 10^{-13}$
22	$4.682\times 10^{-13}$	$4.724\times 10^{-13}$	$4.730\times 10^{-13}$	$4.753\times 10^{-13}$
24	$5.452\times 10^{-13}$	$5.475\times 10^{-13}$	$5.505\times 10^{-13}$	$5.529\times 10^{-13}$
26	$6.570\times 10^{-13}$	$6.630\times 10^{-13}$	$6.630\times 10^{-13}$	$6.689\times 10^{-13}$
28	$6.511\times 10^{-13}$	$6.570\times 10^{-13}$	$6.570\times 10^{-13}$	$6.570\times 10^{-13}$

**Table 6:** Correlation coefficients of the regression model using NOSER algorithm.

Phantom	Regression Model $PV_{max}=aMC-b$	$\mathrm{Correlation}\;\mathrm{Coefficient},\;R^{2}$
A	$PV_{max}=2.113E^{3}MC-2.125E^{4}$	0.9550
B	$PV_{max}=2.115E^{3}MC-2.103E^{4}$	0.9534
C	$PV_{max}=1.954E^{3}MC-1.674E^{4}$	0.9583
D	$PV_{max}=1.915E^{3}MC-1.559E^{4}$	0.9541

**Table 7:** Correlation coefficients of the regression model using Tikhonov Regularization algorithm.

Phantom	Regression Model $PV_{max}=aMC-b$	$\mathrm{Correlation}\;\mathrm{Coefficient},\;R^{2}$
A	$PV_{max}=5.452E^{-14}MC-7.959E^{-13}$	0.9529
B	$PV_{max}=5.502E^{-14}MC-8.039E^{-13}$	0.9536
C	$PV_{max}=5.447E^{-14}MC-7.883E^{-13}$	0.9545
D	$PV_{max}=5.449E^{-14}MC-7.871E^{-13}$	0.9553

**Table 8:** MSSIM Indexes computed on the reconstructed images.

MC (%)	Phantom A		Phantom B		Phantom C		Phantom D	
	NOSER	Tikhonov Regularization	NOSER	Tikhonov Regularization	NOSER	Tikhonov Regularization	NOSER	Tikhonov Regularization
16	0.5221	0.6644	0.3573	0.4161	0.3316	0.3665	0.3118	0.3294
18	0.4912	0.5929	0.3419	0.3577	0.3246	0.3347	0.3091	0.3092
20	0.4596	0.6194	0.3359	0.3873	0.3171	0.3626	0.3040	0.3266
22	0.4477	0.6014	0.3387	0.3835	0.3340	0.3860	0.3189	0.3490
24	0.4673	0.6783	0.3495	0.3933	0.3464	0.4002	0.3308	0.3628
26	0.5349	0.7211	0.3907	0.4776	0.3640	0.4319	0.3497	0.3869
28	0.5389	0.7049	0.3947	0.4589	0.3703	0.4237	0.3558	0.3754

## Data Availability

All data generated in this manuscript are available upon request, with provided approval by the corresponding author. In addition, the models and code used during the research work cannot be shared at present because the data forms part of an ongoing study.
